# Opioid use in Latin America: a vital challenge for health systems

**DOI:** 10.7189/jogh.15.03030

**Published:** 2025-07-01

**Authors:** Alejandra Palma, Pedro E Pérez-Cruz, Katherine Pettus, Tania Pastrana

**Affiliations:** 1 Sección de Cuidados Continuos y Paliativos, Departamento de Medicina Interna Norte, Facultad de Medicina Universidad de Chile, Santiago, Chile; 2 Programa de Medicina Paliativa y Cuidados Continuos, Departamento de Medicina Interna, Facultad de Medicina Pontificia Universidad Católica de Chile, Santiago, Chile; 3 International Association for Hospice and Palliative Care, Houston, Texas, USA; 4 US Department of Palliative Medicine, Uniklinik RWTH Aachen, Aachen, Germany

## Abstract

This viewpoint challenges the notion that Latin America is on the verge of an opioid use disorder crisis similar to that occurring in the USA and Canada. We critically analyse the evidence presented by León and colleagues, who argues that opioid prescribing patterns in countries such as Chile reflect an alarming expansion of misuse, especially in the context of chronic non-cancer pain. While acknowledging the growing availability of opioids in Latin America, we contend that this trend primarily reflects efforts to expand palliative care and rational opioid access for serious health-related suffering. Drawing from regional and national data, we show that increases in opioid consumption in countries like Chile are consistent with public health improvements, including national pain relief programmes and coverage of cancer patients with palliative care services. The evidence of opioid use disorder and non-medical use is limited and does not justify alarmist conclusions. We advocate for balanced strategies that address opioid misuse risks while ensuring access to pain relief, provided they are guided by international standards, data-informed policymaking, and transparent reporting of conflicts of interest. The global health community must prioritise equitable opioid access across Latin America to alleviate serious health-related suffering and uphold the principles of universal health coverage.

In this viewpoint, we challenge Dr León and colleagues’ paper, ‘Opioid use in Latin America: chronicle of a death foretold?’ [1], which asserts that the crisis of opioid use disorder (OUD) observed in Canada and the United States is becoming a global challenge. The authors contend that a similar trend of opioid misuse is emerging in Latin America (LA), particularly in Chile, and conclude that it is important to ‘prevent (…) uncontrolled expansion of opioid prescriptions in LA, and a crisis similar to that experienced in other regions’. They base their concerns on claims of expanded opioid consumption and reportedly high OUD rates in the region. Here we discuss these claims, contributing relevant reflections on the availability and consumption of medical opioids in LA in general, and in Chile in particular.

## OPIOID CONSUMPTION

While we acknowledge that opioid availability in the region has improved in recent years, we challenge León and colleagues’ description of an ‘alarming expansion of opioid prescriptions’ in LA, which focusses primarily on management of chronic non-cancer pain (CNCP). This focus ignores other medical contexts where rational prescription of opioids is crucial, such as in surgery, trauma medicine, palliative care (PC), obstetrics-gynaecology, and treatment of mental health disorders. León and colleagues’ narrow focus on CNCP distorts their analysis of the opioid landscape in LA.

Concerns about harms that flow from misuse of opioids by certain affected populations must be understood in the broader public health context of rational access to opioids in the disciplines mentioned above. We situate rational use within the conceptual framework of serious health-related suffering (SHS) developed by the Lancet Commission on Palliative Care and Pain Relief [2], within which SHS is defined as suffering associated with illness or injury, which becomes serious when it cannot be relieved without medical intervention and when it compromises physical, social, or emotional functioning. Over 25.5 million people (45% of all deaths) experienced SHS in 2015, while more than 80% of those deaths occurred in low- and middle-income countries. Severe pain and dyspnoea were the primary SHS triggers [2].

Experts calculated the rate of SHS in LA to be 5.45 people per 1000 population, or four million people with SHS each year, amounting to 290 million days of pain [3]. There is marked heterogeneity between countries in the region: medical opioid availability in Haiti and Bolivia covers only 1% and 6%, respectively, of the required morphine equivalents for pain relief, while Brazil meets 73% and Argentina 114% of their estimated needs [3].

The number of people in Chile experiencing SHS has nearly doubled since 1990. An estimated 104 923 people experienced SHS in 2019, including 47 000 (44%) cancer patients. Of these SHS, 72% were attributed to physical symptoms, primarily pain. Notably, 93% of cancer patients with SHS in Chile had access to PC, including opioid analgesics [4]. León and colleagues analogised the significant increase in opioid consumption in Chile since 2000 with OUD trends in high-income countries, analysing this pattern of opioid prescription mainly in the context of CNCP, without considering the need for SHS relief by those with oncologic or life-limiting conditions. Chile has developed PC services, as well as increased opioid prescribing capacity with the help of reforms that ensure effective pain management. The country’s National Program for Pain Relief and Palliative Care for advanced cancer patients was established in 1994 and subsequently expanded in 2014 to guarantee public funding for cancer pain relief, regardless of the disease's stage or prognosis [5,6].

León and colleagues also claim that Chilean opioid consumption is ‘far surpassing 200 S-DDD per million inhabitants per day, established by the WHO as the dose level sufficient to relieve pain in all areas’. This statement is misleading: the article referenced in their viewpoint acknowledges the lack of justification for this benchmark, stating that the 200 S-DDD level corresponds to 3.4% of adequate consumption for 2010, which is classified as very low consumption [6]. Chile currently has a distributed opioid morphine equivalent of 2.404 mg/patient, a measure that captures the total national morphine equivalent needed for patients with PC needs. This currently covers 114% of the calculated population need, which is below the recommended benchmark following the methodology proposed by the Lancet Commission in Pain Control and PC [2,3].

The increase in opioid availability observed in Chile over the last decades represents a significant public health achievement that should not be misinterpreted as a failure related to improper opioid use. Furthermore, the need for rational medical opioid prescribing is projected to increase in the coming decades. Population-level SHS associated with PC needs in Chile is projected to increase from 117 000 in 2021 to 209 000 people in 2050. This represents a 79% increase (incidence risk ratio = 1.79; 95% confidence interval = 1.78–1.80) and approximately 75 400 new cancer patients, requiring an increase in opioid analgesics necessary to achieve adequate pain control [7].

## OPIOID USE DISORDERS

León and colleagues assert a trend toward opioid misuse in LA based on two studies. The first reports a 29% prevalence of OUD in a sample of 120 Chilean patients with CNCP, highlighting a higher risk among amputees and those with a history of alcohol use or mental health conditions [8]. The second corresponds to the National Drug Survey report conducted in Chile between 2016 and 2018, which León and colleagues use to support their claim that ‘the number of opioid analgesics consumed without a medical prescription quadrupled in the last decade’ [9]. However, this claim is misleading, as the report indicates that the prevalence of non-medical opioid use was 1% in 2002 and 2.5% in 2018, which does not support the assertion of a four-fold increase. The latest National Drug Survey performed in Chile in 2022–23, which included a representative sample of 17 454 people, shows that the current number of opioid analgesics consumed without medical prescription fluctuated between 1.2 and 1.5%, remaining relatively stable and at low levels overall in recent years. In the same survey, the highest perceived risk of substance use corresponds to the consumption of alcohol, marijuana, and cocaine [10].

Finally, León and colleagues claim that the mortality rate related to opioid use disorder in Chile is low – estimated at 0.036 per 100 000 inhabitants in 2020 – but sound an alarm anyway, suggesting that health authorities should take the steady increase in opioid use, overdose rates, and mortality over the last 20 years seriously. They base this warning on an analysis of aggregate mortality related to several opioid classifications under International Classification of Disease codes T40.0 (opium), T40.1 (heroin), T40.2 (other opioids: codeine, morphine, oxycodone), T40.3 (methadone), T40.4 (fentanyl and pethidine), and T40.6 (unspecified), making it challenging to attribute opioid use disorder to the rising trends of rational clinical use of medical opioids. Due to the limited data on both the levels of OUD and the associated mortality, we disagree with the authors’ alarmist interpretations.

## CONCLUDING COMMENTS

We understand León and colleagues’ apprehensions regarding the potential public health risk associated with OUD, especially in the management of CNCP. The devastating mortality rate resulting from OUD in Canada and the USA should not be ignored. It reflects multifactorial social, regulatory, and political problems that may or may not be reproduced in other countries. However, the authors’ interpretation of increased opioid consumption in LA appears biased, as it primarily considers the risks of opioid misuse in the context of CNCP. This bias overlooks other relevant patient groups, such as those with PC needs who require effective SHS relief, including pain management. As previously noted, the increase in opioid consumption in LA at least partly reflects the successful implementation of PC services and expanded access to medical opioid prescriptions, including morphine and other essential medications listed on the World Health Organization’s List of Essential Medicines [11].

Experts recognise concerns about the addictive potential of opioids, often termed ‘opiophobia’, as one of several barriers limiting rational access to pain relief worldwide. These barriers, including opiophobia, can be effectively addressed through adequate medical education and professional PC and harm reduction programmes. It is also essential to address industry influence and conflicts of interest, including in medical education on opioid prescribing. Scientific societies and academic programmes that produce evidence-based recommendations supported by policies requiring transparency regarding conflicts of interest should lead this education. Unified prescription systems that monitor restricted medications in compliance with international standards without imposing unnecessary obstacles for timely opioid prescription, also contribute to balancing rational opioid access with risk assessment and monitoring. Finally, it is essential to strengthen public education campaigns on the benefits and safe use of opioids, as part of a community-based approach to tackling the multidimensional challenges of opioid prescribing across diverse cultural contexts.

Efforts to overcome barriers to pain relief for cancer and other conditions remain a vital challenge for health systems in LA. The global health community has a duty to help narrow the access abyss in the relief of pain and other sources of suffering caused by life-limiting and life-threatening health conditions.
